# The relationship between balance control and thigh muscle strength and muscle activity in persons with incomplete spinal cord injury

**DOI:** 10.1038/s41394-024-00620-x

**Published:** 2024-02-28

**Authors:** Matthijs Ferdinand Wouda, Marte Fosvold Løtveit, Espen Ingvald Bengtson, Vegard Strøm

**Affiliations:** 1 Sunnaas Rehabilitation Hospital, Nesoddtangen, Norway; 2 Department of Rehabilitation Science and Health Technology, Oslo Metropolitan University, Oslo, Norway; 3 Department of Physical Performance, Norwegian School of Sport Sciences, Oslo, Norway

**Keywords:** Neurophysiology, Spinal cord diseases

## Abstract

**Study design:**

Cross-sectional study.

**Objectives:**

A spinal cord injury (SCI) can compromise the ability to maintain sufficient balance control during activities in an upraised position. The objective of the study was to explore the relationship between balance control and muscle strength and muscle activation in the lower extremities in persons with incomplete SCI (iSCI).

**Setting:**

Sunnaas Rehabilitation Hospital, Norway.

**Methods:**

Thirteen men and two women with iSCI and 15 healthy, matched controls were included. Performance of the Berg Balance Scale (BBS) short version (7 items) was used to indicate balance control. Maximal voluntary contraction (MVC) was performed to measure isometric muscle strength in thigh muscles (knee extension/flexion), while surface electromyography (EMG) was measured from M. Vastus Lateralis and M. Biceps Femoris. The relative activation of each muscle during each of the BBS tasks was reported as the percentage of the maximal activation during the MVC (%EMG_max_).

**Results:**

The iSCI participants had a significantly lower BBS sum score and up to 40% lower muscle strength in knee- flexion and extension compared to the matched healthy controls. They also exhibited a significantly higher %EMG_max_, i.e. a higher muscle activation, during most of the balance tests. Univariate regression analysis revealed a significant association between balance control and mean values of %EMGmax in Biceps Femoris, averaged over the seven BBS tests.

**Conclusions:**

The participants with iSCI had poorer balance control, reduced thigh muscle strength and a higher relative muscle activation in their thigh muscles, during balance-demanding activities.

## Introduction

Close to half of all spinal cord injuries (SCI) are functionally incomplete [1, 2], which implies some remaining motor or sensory function below the level of the lesion [3]. These injuries can significantly impact an individual’s ability for independent locomotion [1]. Regaining locomotor function after an incomplete spinal cord injury (iSCI) is of high priority for the affected individuals [4], and individuals with these injuries have a great possibility to regain some walking function after rehabilitation [1, 5]. While many individuals with iSCI relearn to walk [1], there is a high rate of falls in this population [6] that can lead to further injuries and hospitalization and may lower their confidence and community participation [7, 8]. Balance control requires the integration of sensory input and motor output. Sensorimotor impairments following an iSCI can compromise the ability to maintain sufficient balance control during locomotion [9].

Possibly, there are extensive mechanisms behind the limitations in maintaining balance for individuals with iSCI [10]. Factors that may affect the ability to maintain balance are skeletal muscle strength, pain, and loss of motor and/or sensory function [11]. With a partial loss of motor function and/or sensory input below the level of injury, reduced neural signals to affected muscles can produce varying skeletal muscle activation, which can affect balance control [12, 13]. In addition, it may indicate several clinically relevant motor and functional deficits, including local muscle fatigue, weakness in affected muscles [14, 15], and a reduced ability to perform various movements [4, 16]. This may result in reduced walking speed, limited range of motion [4], and reduced coordination in both upper and lower extremities, which could limit their ability to stand and walk [17]. However, exercise has been shown to preserve muscle mass [18] and restore motor and sensory function [19, 20], as increased skeletal muscle strength correlates with both greater balance and gait ability for individuals with iSCI [21, 22].

The force generated by a skeletal muscle depends on numerous factors, including the degree of activation of the nervous system, its architecture, muscle size, the space between myofilaments, the number of actin-myosin cross-bridges formed, the force generated by each cross-bridge and the quality of the interaction between the cellular elements [23]. The force in a muscle is regulated by the number of motor units recruited, and the power developed in the activated units. The force in each motor unit is controlled by the frequency of the action potentials that reach the linked muscle fibers [24].

The degree of muscle activation in the lower-extremity muscles might be associated with balance control in persons with iSCI [25]. Surface electromyography (sEMG) is a technique measuring muscle activation by the strength of the electrical signal produced by activating individual motor units. Enhanced sEMG analysis could contribute to a more complete description of the effects of SCI on motor neuron function and their interactions, and it might assist in understanding the mechanisms of change following neuromodulation or exercise therapy [26]. The aim of the present study was to describe balance control, muscle strength and muscle activation in the lower extremities, and their correlates, in persons with iSCI.

## Methods

### Design

This cross-sectional study included 15 participants with an incomplete SCI and 15 matched healthy controls. The study was approved by the Regional Committee for Medical and Health Research Ethics (Ref: 352478) and the Norwegian Centre for Research Data (Ref.: 637499).

### Participants

Between January 2022 and April 2022, 13 men and two women who received inpatient rehabilitation at Sunnaas Rehabilitation Hospital were included according to the following inclusion criteria: incomplete SCI (AIS C or D), >2 weeks post-injury, between 18 and 75 years of age and being able to stand without support. In addition, healthy controls (*n* = 15), matched on age, sex, and Body Mass Index (BMI) were recruited among employees at the hospital. Participants were excluded if they had medical conditions limiting their physical capacity, e.g., psychiatric conditions, orthostatic hypotension, and severe activity-induced pain in the lower extremities.

### Procedures

After giving written informed consent, a medical approval was given by a specialist in physical medicine and rehabilitation. All participants were asked to refrain from alcohol, exercise, and strenuous physical activity 24 h prior to the tests. Upon arrival at the laboratory on the day of testing, surface electrodes for measuring electromyography (EMG) were mounted on the participants’ thigh musculature. Thereafter they performed the following tests in a fixed order: a maximal isometric knee extension/flexion test, a sitting leg-press test, the Berg Balance Scale (BBS) test, the 10-m Walk Test (10MWT), and the Timed Up and Go (TUG) test. The 10MWT and TUG were not performed by the healthy controls. In addition to the clinical tests, the SCI participants completed the Spinal Cord Independent Measure III (SCIM III) questionnaire [27]. The participants used approximately 1.5 h to complete all tests. The injury characteristics, including time since injury, AIS score, including lower-extremity motor score (LEMS) and, upper extremity motor score (UEMS), Traumatic injury (T), and non-traumatic injury (NT), were retrieved from the electronic medical records before clinical testing was performed.

### Measurements

#### Electromyography

In preparation for EMG-measurements, hair was removed from the selected skin areas if necessary, skin abrasion was performed, cleaned (alcohol wipe) and then air-dried.

**Fig. 1:**
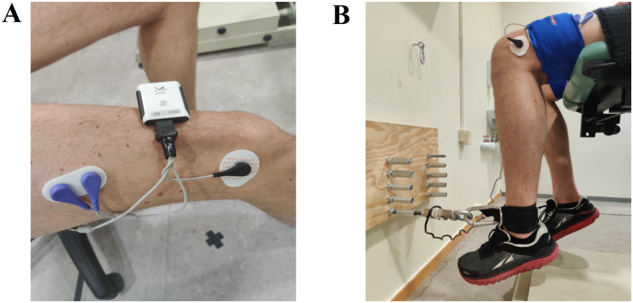
EMG placement and maximal isometric muscle strength testing setup. **A** Placement of the EMG electrodes (VL). **B** Attachment of the inelastic ankle strap to the force sencor.

Surface EMG dual electrodes (Dymedix Diagnostics, Shoreview, MN, USA), with 2.5 cm separation, were attached along the direction of the muscle fibers on the M. Vastus Lateralis (VL) and M. Biceps Femoris (BF). The VL-sensors were placed at 2/3 on the line from the anterior spina iliaca superior to the lateral side of the patella, while the BF-sensors were placed ½ of the line between the ischial tuberosity and the epicondyle of the tibia (see Fig. 1A). The surface electrodes were attached with a double channel EMG cable to a wireless EMG module (Muclelab, Ergotest Innovation AS, Stathelle, Norway). Both the EMG cables and EMG module were held in place to the thigh by Velcro bands (see Fig. 1B).

#### Maximal voluntary isometric contraction

Participants were positioned in an individually adjustable chair. An inelastic ankle strap was mounted just above the lateral malleolus and attached to a force sensor (Muclelab, Ergotest Innovation AS, Stathelle, Norway) that was fixed to the wall (see Fig. 1B). The hip and chest were fixed to the chair with a four-point seatbelt. The participants performed one test-trial at submaximal effort and three trials at maximal effort of unilateral knee flexion and extension, under encouragement of the evaluator. Between each trial there was a 1-min break. The trials were performed in a standardized order; flexion of the right knee, extension of the left knee, extension of the right knee, and finally flexion of the left knee. All unilateral trials were performed with 90° knee flexion and 90° hip flexion. The maximum force (Newton; N) generated by the participants were recorded for each trial, and the trial with the highest measured EMG signal (EMG_max_ (microvolt; µV)) was regarded as the maximal voluntary contraction (MVC). After having performed the unilateral trials, participants performed a seated leg-press with an integrated force plate, to measure their maximal isometric muscle strength during leg-press. The seat angle and distance to the force plate were adjusted so that both the knee and hip angle was 75°. During leg-press testing, EMG was not measured.

#### Balance

BBS was used to measure the participants balance control in upright position. The BBS originally includes 14 items; 1 item on balance when sitting and 13 items on balance when standing [28]. In our study, we used the BBS *short version* [29], which includes a 7-item list. The seven tasks were performed, with 30–60 s rest in between, in this order: sitting to standing (BBS 1), standing with eyes closed (BBS 2), reaching forward outstretched (BBS 3), retrieving an object (a cup) from the floor (BBS 4), standing while turning to look behind (BBS 5), standing with one foot in front (BBS 6), and standing on one foot (BBS 7). Each item was scored on a five-point ordinal scale ranging from 0 to 4, with 0 indicating the lowest level of function and 4 the highest level of function [29]. Adding up the item scores gave a total BBS score, ranging from 0–28. Muscle activation in VL and BF was measured during the performance of each item.

#### Functional tests

To assess the participants’ functional mobility and gait, the 10MWT and TUG were performed in accordance with established procedures [30, 31]. Both tests are valid and reliable measures for assessing walking function in persons with SCI [32, 33]. The results were compared to established reference values for 10MWT [34], and TUG [35], respectively.

#### Data processing

The EMG signal was sampled at a frequency of 1000 Hz and was automatically processed using a 20–500 Hz band-pass filter. Additionally, during the signal processing there was calculated a 50-points moving average of the absolute values of each signal to level the peaks, using a custom-made VBA-script made in Microsoft Excel 2016 (Microsoft, Washington, USA). The highest moving average of the EMG signal (µV) during the three tests of the MVC for VL (extension of the knee) and BF (flexion of the knee), respectively, was calculated and used as a baseline to compare the relative muscle activation during the BBS. The relative muscle activation (%EMG_max_) for the VL and BF, both left and right, was calculated for the seven BBS items separately and summarized. In addition, the area under the EMG curve during a one-second timeframe with the highest amplitude during BBS tests 2 and 7 was calculated, and compared to iEMG_max_ to estimate the relative amount of muscle activation over time, i.e. %iEMG_max_. BBS 2 and 7 were used as these tests can be regarded as static balance tasks, and therefore more suitable for estimating %iEMG_max_.

#### Statistical analysis

Statistical analyses were performed with SPSS (IBM Corp. Released 2021. IBM SPSS Statistics for Windows, Version 28.0. Armonk, NY: IBM Corp). All data are reported as mean and standard deviation (SD) unless otherwise stated. For all tests, statistical significance was set at an alpha level of 0.05. Independent sampled t-tests or Mann-Whitney U tests were used to describe differences between the SCI group and the matched control group. To describe the relationship between the dependent variable BBS sum score and the independent variables (i.e. age, BMI, time since injury, muscle activation (%EMG_max_ and %iEMG_max_) and muscle strength) a univariate linear regression analysis were performed. Correlation coefficients indicated no (0 to ±0.1), low (±0.1 to ±0.3), moderate (±0.4 to ±0.6), or strong (±0.7 to ±1.0) correlation [36].

## Results

Table 1 depicts the demographics and injury-specific characteristics of each of the iSCI participants (*n* = 15). The mean age was 52 (15.4) years, the mean height 182 cm (6.5), the mean weight 88 kg (13.6), and the mean BMI was 27 (3.2). There were no statistically significant differences between the iSCI and control groups in gender, age, or BMI. All participants included in this study performed all the tests. No adverse events were reported. Participants with iSCI used more time (+ 4.63 (4.64) seconds) to perform the 10 MWT (with a mean time of 9.33 (5.14) seconds, and TUG (+ 2.33 (0.63) seconds (with a mean time of 11.30 (7.86) seconds compared to age-adjusted reference values [34, 35].

**Table 1:** Demographic and injury-specific characteristics of the iSCI participants.

ID	Gender	Age	Type of injury	AIS grade	Lesion	Time after injury (months)	LEMS	UEMS	SCIM III	Mobility status (>100 m)
1	M	20–25	NT	D	Th7-Th11	198	36	50	69	Walks without walking aids
2	M	30–35	T	D	C8	42	50	50	70	Walks without walking aids
3	M	60–65	T	D	Th12	432	40	50	65	Walks with one cane
4	M	40–45	NT	D	Th4	12	50	50	69	Walks without walking aids
5	M	60–65	T	D	C2	174	47	49	70	Walks without walking aids
6	M	20–25	T	D	C4	48	47	44	64	Walks without walking aids
7	M	60–65	T	D	C2	1	50	46	70	Walks without walking aids
8	M	50–55	T	D	L2	432	41	50	69	Walks without walking aids
9	F	60–65	NT	D	C8	121	43	48	70	Walks without walking aids
10	M	55–60	NT	A^a^	L5	88	50	50	70	Walks without walking aids
11	M	45–50	T	A^a^	Th9	84	50	50	54	Electric wheelchair or partial assistance to operate a manual wheelchair
12	M	60–65	T	D	Th1	18	49	49	67	Walks with crutches or two canes
13	M	70–75	T	D	C4	0.8	50	50	69	Walks without walking aids
14	F	50–55	NT	D	Th4	60	40	50	57	Walks with a walking frame or crutches
15	M	65–70	NT	C	Th5	16	45	50	61	Walks with crutches or two canes

*ID* identification, *M* male, *F* female, *LEMS* lower-extremity motor score, *UEMS* upper extremity motor score, *T* Traumatic, *NT* non-traumatic, *C* cervical, *Th* thoracic, *L* lumbar, *S* sacral, *SCIM III* spinal cord independent measure III.

^a^Sensory complete, but motor incomplete spinal cord injury.

### Balance control

The mean BBS sum score for the participants with iSCI was 21 (5.0), ranging from 10–27, which was significantly lower than for those in the control group (*p* < 0.001), in which all except one had a maximum BBS score of 28.

### Muscle strength

**Fig. 2:**
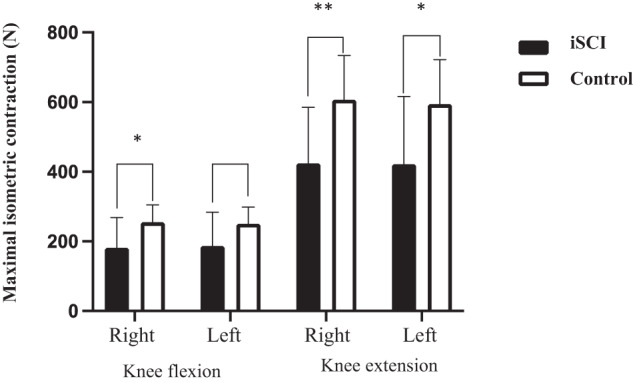
Difference in maximal isometric contraction (N) in knee flexion and knee extension exercise measured, between iSCI participants and controls. iSCI incomplete spinal cord injury; N newton; The number of stars (*) indicates the significance level of the result. Where *p < 0.05 and **p < 0.01.

Figure 2 shows that the MVC was significantly different between the iSCI group and the control group for both right and left knee extension (resp. *p* = 0.004 and *p* = 0.021) and for right knee flexion (*p* = 0.026), while it did not reach statistically significance in the knee flexion of the left leg (*p* = 0.057). The control group had an average of 34% higher force (N) in knee flexion and 40% higher force (N) in knee extension than the iSCI group (Fig. 2).

In addition, we found a significantly lower maximal leg-press strength (*p* = 0.01), in the participants with iSCI (1262 N ±542), compared to the control group (1697 N ±106).

### Muscle activation - %EMG_max_ and %iEMG_max_

**Fig. 3:**
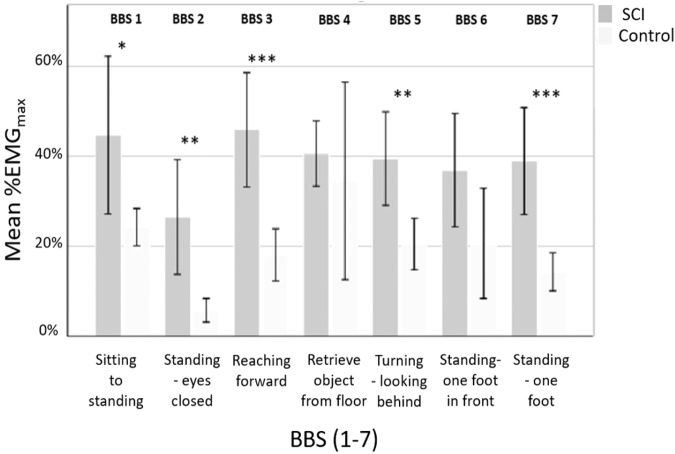
Percent of maximal EMG (%EMG_max_) during the 7 items Berg Balance Scale (BBS) tests for the iSCI group (*n* = 13) and the control group (*n* = 14). The bars represent mean values and 95% CI of averaged %EMG_max_ values for all four thigh muscles (BF and VL, left and right). For example, participants with iSCI, when standing on one foot (BBS 7), used almost 40% %EMG_max_, while healthy controls used approximately 13% %EMG_max_ during the same activity. iSCI incomplete spinal cord injury. The number of stars (*) indicates the level of significance: *p < 0.05, **p < 0.01, ***p < 0.001.

Figure 3 presents the mean %EMG_max_ values for the VL and BF muscles of both left and right leg combined during each the seven BBS tests for the iSCI and control groups. There was a statistically significant difference in %EMG_max_ between the iSCI group and the controls in the BBS tests 1 (*p* = 0.027), 2 (*p* = 0.004), 3 (*p* < 0.001), 5 (*p* = 0.003) and 7 (*p* < 0.001), while there was no significant difference in the BBS tests 4 (*p* = 0.587) and 6 (*p* = 0.056) between the groups (Fig. 3).

On average, participants with iSCI used 36% (CI95%: 28–43%) of their EMG_max_ for the VL and 42% (CI95%: 33–52%) for the BF, during BBS 1–7. The control group used on average 22% (CI95%: 10–35%) for the VL and 17% (CI95%: 13–21%) for the BF, during BBS 1–7. Appendix 1 describes in detail the muscle activation (%EMG_max_) of each of the thigh muscles, including the difference between the iSCI and control group, during each of the 7 items of the BBS.

Table 2 shows that during the BBS items 2 (standing with eyes closed) and 7 (standing on one foot), the %iEMG_max_ in both thigh muscles (VL and BF) was significantly higher in the iSCI group compared to the control group.

**Table 2:** Mean differences in %iEMG_max_ between the iSCI and the control group.

Test	Muscle	iEMG_max_		
		p-value	Mean difference	95% CI
BBS 2	VL	0.004	+14%	5.0–22.4
	BF	0.015	+9%	2.1–16.8
BBS 7	VL	0.004	+14%	5.3–23.7
	BF	0.005	+15%	5.2–23.9

*CI* confidence Interval, *BBS* Berg Balance Scale, *VL* Vastus Lateralis, *BF* Biceps Femoris.

### Relationship between muscle activation, muscle strength, and BBS

Table 3 shows the results of the univariate regression analyses of the associations with balance control (i.e. the BBS sum score) in the iSCI participants, revealing only the mean %EMG_max_ of BF, averaged for the left and right side over the seven BBS tests, to reach statistically significance (*p* < 0.001).

**Table 3:** Results of a univariate generalized linear mixed model regression analysis on how age, BMI, time since injury, muscle strength, and muscle activation are associated with BBS in participants with iSCI (*n* = 13)^a^.

Independent variable	Coefficient	*p*-value	95% CI
Age (years)	−0.15	0.10	−0.34 to 0.03
BMI	−0.38	0.40	−1.34 to 0.58
Time since injury (months)	−0.01	0.34	−0.03 to 0.01
Leg-press strength (*N*)	0.004	0.26	−0.003 to 0.01
Average %EMG_max_ VL during BBS 1–7^b^	−0.19	0.12	−0.44 to 0.06
Average %EMG_max_ BF during BBS 1–7^b^	−0.28	<0.001^a^	−0.38 to −0.17

*BMI* Body Mass Index, *%EMG*_*max*_ muscle activation, *VL* Vastus lateralis, *BF* Biceps Femoris, *BBS* Berg Balance Scale.

^a^Two participants were excluded due to lacking BF muscle strength in sitting position.

^b^Averaged %EMG_max_ for left and right side.

## Discussion

The main findings of this study are that the participants with iSCI, compared to healthy matched controls, had significantly lower BBS sum score and lower thigh muscle strength. Participants with iSCI also had significantly higher thigh muscle activation (mean %EMG_max_) in both the left and right leg, in five of the seven BBS tests. Moreover, the iSCI participants’ mean %EMG_max_ of BF averaged over the seven BBS tests, exhibited significant association with the BBS sum score.

The lower balance scores found in the iSCI group compared to the healthy controls might be explained by impaired somatosensory sensation as SCI causes interruption of the sensorimotor processes underlying balance control, with varying degrees of balance deficits as a result [37]. This is supported by our findings of reduced strength of the lower extremities (Fig. 2) and the higher %iEMG_max_ in both thigh muscles (VL and BF) than the controls when performing balance tests standing with eyes closed and standing on one foot (Table 2). Furthermore, those with iSCI performed poorer on the 10MWT and TUG tests which strongly correlated to lower balance scores, consistent with similar studies conducted on individuals with iSCI [21, 22, 38–40].

To our knowledge, this is the first study that has examined the %EMG_max_ during balance-demanding exercise for an iSCI population. While performing the seven BBS tests, those in the iSCI group exhibited 5% to 35% higher relative %EMG_max_ in the VL and BF compared to that in the control group. In addition, the iEMG_max_ in the BF was significantly higher in the iSCI group compared to the control group. However, the mechanisms behind this difference in individuals with iSCI are difficult to determine. This study shows that neither age, time since injury or muscle strength seem to predict BBS sum score in participants with iSCI. The central nervous system obtains information on mechanical and chemical changes through sensory receptors located within muscles and joints. In participants with iSCI this feedback system can partly be damaged, which can lead to reduced balance abilities during weight bearing activities. To compensate, the muscle might activate even more motor units to regain control. However, it has been shown that individuals with iSCI seem to maintain good control of the available motor units, despite a limited number of muscle fibers in their lower leg muscles [41]. Therefore, our results could indicate that individuals with iSCI show a relatively higher effort and activity in their thigh muscles to compensate for the limited number of muscle fibers. Furthermore, the participants with iSCI had on average 34% lower muscle strength in the knee flexion and 40% lower in the knee extension than the controls. Studies comparing lower-extremity muscle maximum cross-sectional area (CSA) between persons with iSCI and a matched controls show smaller average muscle CSA in affected lower-extremity muscles compared with the control participants [13, 42, 43]. As muscle atrophy is strongly related to reduced muscle strength [44, 45], this difference in CSA may support our findings.

The participants with iSCI in this study used up to 50% of %EMG_max_ during some of the balance exercises. It is reasonable to believe that balance-demanding activities in everyday life may be more demanding for people with iSCI as it may lead to higher muscle activation and lead to these persons experiencing muscle fatigue more frequently [14, 15]. Another reason for the high muscle activation in iSCI could be that the knee flexors and extensors must compensate for weaknesses in other distal muscle groups. However, this cannot be confirmed with our results because no iEMG_max_ or strength measurements below the knee joint have been performed in our study. In contrast, we found a tendency towards increased muscle activation in the exercises that place higher demands on stabilization in the ankle joint. For example, participants with iSCI used 20–35% more muscle activity than the control group during BBS item 7, in which they stand on one foot. Reduced sensory information in the feet can also be an inhibiting factor during balance exercises, which can contribute to higher demands on the leg muscles to maintain balance. Several studies show that sensory information plays a critical role in walking ability and movement after SCI [46, 47].

### Methodological limitations

The findings from this study cannot be interpreted fully without accounting for its limitations. The low sample size in this study limits the generalizability of the results.

Muscle activation measurements were, for practical reasons, limited to the knee extensors and flexors. It is assumable that other muscles around the, hip and ankle joints contributed extensively during BBS testing of the participants. Another limitation in this study is that BBS has a clear ceiling effect, which is reflected in the fact that the participants with apparently impaired balance got a higher score than expected during the testing. There is little to no consensus in the research literature on which test should be used to obtain a measure of balance for people with SCI due to the BBS’s reported ceiling effect [40], as it apparently cannot distinguish between individuals with iSCI who have different ambulatory balance abilities. In addition, BBS has not been able to predict falls for SCI populations [22, 40, 48, 49]. In contrast, our study did not demonstrate a ceiling effect, described as >20% of participants achieving the maximum score on the scale [50]. However, the purpose of our study was not to compare scores for the participants with SCI and the control group to validate the BBS.

### Clinical implications

This study has provided new knowledge on muscle activation during balance-demanding exercises in persons with SCI. The relatively high muscle activation during balance testing in the participants with iSCI, shows the importance of sufficient thigh muscle strength to maintain functional mobility in this patient group. The study results may be valuable for clinicians, as it may lead to a more specific rehabilitation program for people with iSCI, who have standing and/or gait function. However, future studies should include a larger sample and should include the measurement of %EMG_max_ and strength of more muscle groups simultaneously. It may also be interesting to look at differences in %EMG_max_ compared to other patient groups with approximately the same pathophysiology.

In conclusion, this study described muscle strength during knee flexion- and extension, and muscle activation during BBS testing in persons with iSCI and healthy, matched controls. The participants with iSCI had poorer balance control, reduced thigh muscle strength and a higher relative muscle activation in their thigh muscles, during balance-demanding activities.

### Supplementary information

Appendix A

## Data Availability

Additional data are available from the corresponding author on reasonable request.
