# Supplementary material for: The relationship between balance control and thigh muscle strength and muscle activity in persons with incomplete spinal cord injury
Source: Spinal Cord Ser Cases. 2024 Feb 28;10:7. doi: 10.1038/s41394-024-00620-x (PMC10902359; doi:10.1038/s41394-024-00620-x)
Supplement: Supplementary file 1 — Appendix A [file 41394_2024_620_MOESM1_ESM.docx]

**Appendix 1**. Muscle activation (%MVC), including between-group differences, during the 7-item BBS for

M. Biceps femoris and M. Vastus lateralis.

| BBS | Muscle | Right/ Left | % of MVC  SCI | % MVC  Control | Between groups difference  (%) | p-value | 95 % confidence interval | |
| --- | --- | --- | --- | --- | --- | --- | --- | --- |
|  |  |  |  |  |  |  | Lower | Upper |
| Sitting to standing | Biceps femoris | Right | 46% | 20% | +26% | 0.001** | 12 | 40 |
|  |  | Left | 40% | 35% | +5% | 0.800 | -34 | 44 |
|  | Vastus lateralis | Right | 65% | 45% | +19% | 0.033* | 2 | 37 |
|  |  | Left | 53% | 39% | +15% | 0.0056* | -0.4 | 30 |
| Standing with eyes closed | Biceps femoris | Right | 28% | 6% | +22% | 0.012* | 6 | 38 |
|  |  | Left | 30% | 12% | +18% | 0.130 | -6 | 42 |
|  | Vastus lateralis | Right | 24% | 6% | +18% | 0.004** | 6 | 30 |
|  |  | Left | 24% | 5% | +20% | 0.005** | 7 | 32 |
| Reaching forward with outstretched arm | Biceps femoris | Right | 56% | 23% | +33% | 0.002** | 14 | 51 |
|  |  | Left | 47% | 26% | +21% | 0.085 | -3 | 46 |
|  | Vastus lateralis | Right | 37% | 13% | +26% | <0.001*** | 13 | 39 |
|  |  | Left | 41% | 16% | +25% | 0.016* | 5 | 45 |
|  | Biceps femoris | Right | 39% | 20% | +19% | 0.018* | 4 | 35 |
|  |  | Left | 35% | 26% | +9% | 0.376 | -11 | 28 |
|  | Vastus lateralis  Retrieving object from floor | Right | 48% | 66% | -19% | 0.637 | -98 | 61 |
|  |  | Left | 41% | 30% | +11% | 0.144 | -4 | 27 |
| Turning to look behind | Biceps femoris | Right | 42% | 26% | +15% | 0.089 | -2 | 33 |
|  |  | Left | 51% | 32% | +19% | 0.146 | -7 | 45 |
|  | Vastus lateralis | Right | 34% | 17% | +17% | 0.016* | 3 | 31 |
|  |  | Left | 31% | 13% | +18% | <0.001*** | 9 | 26 |
| Standing with one foot in front | Biceps femoris | Right | 42% | 14% | +28% | 0.004** | 11 | 45 |
|  |  | Left | 35% | 21% | +14% | 0.160 | -6 | 35 |
|  | Vastus lateralis | Right | 37% | 40% | -3% | 0.887 | -49 | 43 |
|  |  | Left | 33% | 13% | +20% | 0.013* | 5 | 36 |
| Standing on one foot | Biceps femoris  Vastus lateralis | Right | 425 | 18% | +24% | 0.007** | 7 | 40 |
|  |  | Left | 50% | 15% | +35% | 0.001** | 16 | 54 |
|  |  | Right | 37% | 11% | +26% | <0.001*** | 13 | 39 |
|  |  | Left | 27% | 7% | +20% | 0.005** | 8 | 39 |

*BBS Berg balance scale; MVC maximal voluntary muscle contraction; The number of stars (*) indicates the significance level of the results. Where (*) p < 0.05, (**) p < 0.01, (***) p < 0.001.*
